# Worth Knowing

**Published:** 1885-09

**Authors:** 


### WELL WORTH KNOWING AND REMEMBERING.

There are few drugs more frequently prescribed in combination now a-days than bromide potassium and chloral hydrate. In fact, there is no better hypnotic to be found than equal parts of those two salts in solution. They constitute, with a little extract cannabis and extract hyoscyamus, the very popular bromidia. It is not generally known that it is dangerous to prescribe them in a solution which contains any alcohol whatever. If put up in a mixture containing even a few drachms of paregoric, the chloral hydrate is converted into the alcoholate, which is harsher and stronger, and which separates and floats on top; and unless very particular instructions are given to shake the vial, there is danger in a patient getting a fatal dose. There is quite a long article on the subject in the *Polyclinic*.

